# Impact of Environmental Pollutants on Gut Microbiome and Mental Health via the Gut–Brain Axis

**DOI:** 10.3390/microorganisms10071457

**Published:** 2022-07-19

**Authors:** Samradhi Singh, Poonam Sharma, Namrata Pal, Manoj Kumawat, Swasti Shubham, Devojit Kumar Sarma, Rajnarayan R. Tiwari, Manoj Kumar, Ravinder Nagpal

**Affiliations:** 1 National Institute for Research in Environmental Health, Bhopal 462030, India; sammradhisingh@gmail.com (S.S.); poonam.mannan91@gmail.com (P.S.); namratapal017@gmail.com (N.P.); manojkbiochem@gmail.com (M.K.); swasti.shubham@gmail.com (S.S.); dkbiotek@gmail.com (D.K.S.); rajtiwari281@yahoo.co.in (R.R.T.); 2 Department of Nutrition and Integrative Physiology, Florida State University, Tallahassee, FL 32302, USA

**Keywords:** mental health, gut microbiota, gut–brain axis, gut dysbiosis, environmental pollutants

## Abstract

Over the last few years, the microbiome has emerged as a high-priority research area to discover missing links between brain health and gut dysbiosis. Emerging evidence suggests that the commensal gut microbiome is an important regulator of the gut–brain axis and plays a critical role in brain physiology. Engaging microbiome-generated metabolites such as short-chain fatty acids, the immune system, the enteric nervous system, the endocrine system (including the HPA axis), tryptophan metabolism or the vagus nerve plays a crucial role in communication between the gut microbes and the brain. Humans are exposed to a wide range of pollutants in everyday life that impact our intestinal microbiota and manipulate the bidirectional communication between the gut and the brain, resulting in predisposition to psychiatric or neurological disorders. However, the interaction between xenobiotics, microbiota and neurotoxicity has yet to be completely investigated. Although research into the precise processes of the microbiota–gut–brain axis is growing rapidly, comprehending the implications of environmental contaminants remains challenging. In these milieus, we herein discuss how various environmental pollutants such as phthalates, heavy metals, Bisphenol A and particulate matter may alter the intricate microbiota–gut–brain axis thereby impacting our neurological and overall mental health.

## 1. Introduction

The gut microbiome is made up of approximately 100 trillion miсroorgаnisms that collectively have almost 200 times more genes than the human genome, making it an “organ” in and of itself [1,2,3,4]. Bасteroidetes аnd Firmiсutes are the two main bacterial phyla that dominate the human intestine, accounting for 90% of intestinal bacteria in healthy people according to findings based on gene sequencing [5]. Proteobacteria, Actinobacteria, Verrucomicrobia and Fusobacteria, among others, make up the remaining 10% [5]. The gut microbiota distribution differs greatly between individuals and even changes throughout life. The co-evolution of human beings and their microbiota has emerged due to a symbiotic interplay and сo-deрendenсy for both species’ existence, ensuing in biomolecular networks between them [6,7]. Bacterial populations in this state are constantly changing, and they are vulnerable to changes in the host environment and body conditions. The inflammation and disruption of gut permeability appear to be caused by gut dysbiosis that, in turn, can have an impact on the host’s health [8]. Gut dysbiosis caused by environmental pollutants leads to alterations in the GBA, which is linked to the onset or exacerbation of psychiatric disorders [9].

Mental health is very important at all stages of life, from childhood through adolescence to adulthood, and impacts how a person behaves, feels and thinks [10]. According to the World Health Organization (WHO), mental health and drug addiction disorders have increased by 13 percent in the last decade (to 2017), owing primarily to demographic shifts [11]. Approximately 450 million people worldwide suffer from some type of psychiatric disorder that accounts for the loss of around one-third of the disability-free-life years [12]. Currently, mental ailments are a prominent cause of disability and morbidity worldwide [13]. Depression, anxiety, phobias, bipolar disorder, schizophrenia and other psychoses, dementia, and developmental disorders such as autism and post-traumatic stress disorder are all mental disorders with their own set of symptoms. A combination of aberrant thoughts, perceptions, emotions, behavior and interpersonal relationships characterize these disorders [14].

Many variables can increase the chance of having a mental disease, but most of them are caused by a combination of environmental, psychological and biological factors. Genetics, brain injury, microbial infection, substance abuse, poor nutrition or exposure to environmental pollutants all may play an important role in the development of mental disorders [15]. To this end, this review discusses different routes that connect microbiota, the gut and the brain, while summarizing recent research pertaining to the effects of environmental pollutants such as heavy metals, phthalates, Bisphenol A (BPA), particulate matter (PM) and others on microbiota. In addition, we deliberate the significance of early exposures on gut dysbiosis and developmental neurotoxicity while pondering some microbiota-targeted intervention strategies which may aid in alleviating mental disorders.

## 2. The Microbiota–Gut–Brain Axis

The bidireсtionаl сommuniсаtion between the enteric nervous system (ENS) and the central nervous system (CNS), linking peripheral digestive activities to the brain’s emotional, behavioral and cognitive centers constitutes the gut–brain axis (GBA) [16]. Much of the early investigations on gut–brain communication concentrated on digestive function [17,18], but more recent studies have focused on the higher-order psychological and intellectual effects of brain–gut and gut–brain communication [19,20,21].

**Figure 1:**
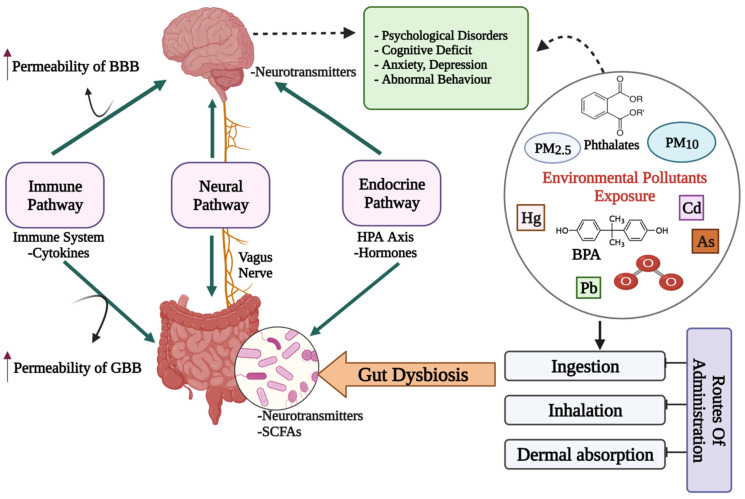
A diagrammatic representation of the putative bidirectional connections regulating the microbiota–gut–brain (MGB) axis. BBB: Blood–Brain barrier; GBB: Gut–Blood barrier; HPA axis: Hypothalamus–Pituitary–Adrenal axis; SCFAs: Short chain fatty acids; Cd: Cadmium; As: Arsenic; O_3_: Ozone; BPA: Bisphenol A; PM: Particulate matter; ↑: increased/higher.

The microbiota has been the focus of research in recent years to discover a missing link between mental health and gut dysbiosis. More than 98% of bacteria in the human gut come from four phyla, viz. Bacteroides, Firmicutes, Proteobacteria and Actinobacteria, making up the microbiome’s remarkable complexity and diversity. The human gut microbiome may comprise more than 1000 bacterial species, containing more than nine million genes, according to metagenomic studies [22]. The presence of a microbiota–gut–brain axis (MGBA) is indicated by the fact that changes in the composition and amount of gut microbes via diet, host-derived metabolites and different environmental contaminants, can affect both the CNS and the ENS [16,23]. The engagement between microbiota and the CNS is principally mediated by the neurological (ANS), hormonal (HPA axis) and immunological (cytokine and chemokine) pathways, which are all linked [20]. Gut microbiome dysbiosis caused by exposure to environmental pollutants may be a direct factor that affects the GBA’s normal functioning and contributes to mental health issues such as depression, anxiety and mood disorders [24] (Figure 1).

### Crosstalk between Microbiota, Gut and Brain

Although the precise processes underlying microbiota–gut–brain crosstalk are as yet unknown, there are several potential mechanisms through which the gut bacteria can alter brain function. Microbes can influence CNS processes bidirectionally via the vagus nerve [25]; through immune system modulation [26]; regulating the activity of the HPA axis, including the plasma level of glucocorticoids [27,28]; tryptophan metabolism [29]; production, expression and turnover of neurotransmitters and neurotrophic factors [30,31,32]; and production of metabolites with neuroactive properties, such as short-chain fatty acids (SCFAs) [31,33,34,35,36]. The gut microbiota imbalance has been proven in animal studies to affect brain chemistry, metabolic status and neuronal function [23]. SCFAs can cross the blood–brain barrier (BBB) via monocarboxylate transporters (MCTs) by overexpressing tight junction proteins and maintaining the integrity of the BBB [36]. SCFAs such as propionate, butyrate and acetate may modulate the levels of neurotrophic factors (BDNF), promote neurogenesis, influence glial cell morphology and function, contribute to serotonin formation and improve neuronal homeostasis and function, all of which help to regulate neuroinflammation in the CNS [36]. The engagement of SCFAs with these gut–brain networks can alter cognition, emotion and the pathophysiology of mental disorders directly or indirectly [36]. Changes in neurotransmitter activity via modulatory pathways including the kynurenine pathway [37], as well as changes in the availability and effects of SCFAs in the brain, can all have an impact on brain-derived neurotrophic factor (BDNF) functions including neuronal survival and differentiation in the CNS [37]. SCFAs also alter the release of gut hormones such as peptide tyrosine tyrosine (PYY), cholecystokinin (CCK) and glucagon-like peptide-1 (GLP-1) from gut mucosal enteroendocrine cells expressing free fatty асid receptors (FFАRs) [38]. In rodents, blood-borne PYY and GLP;-1 permeate the brain and have significant effects on neurotransmitters and behavior [39,40,41]. Daily exposure to the diverse variety of environmental pollutants affects our gut microbiota and impairs the bidirectional GBA and may lead to the development of psychiatric disorders [23]. Manipulation of the intestinal microbiota using prebiotics, probiotics or antimicrobial drugs is an effective therapeutic or preventative measure to counteract behavioral and cognitive deficits and these may be useful to supplement the action of drugs in the treatment of CNS disorders.

## 3. Impact of Environmental Pollutants on Gut Dysbiosis and Mental Health

The various environmental contaminants produced by modern civilization has expanded considerably as industrial processes and technology have advanced around the world. Although the impact of pollution on public health is well documented, little is known about the link between environmental pollutants, gut dysbiosis and mental health [42]. Host diseases (immunological, gastrointestinal and neurobehavioral) can arise as a result of changes in the microbiota that favor more pathogenic organisms producing virulence factors like lipopolysaccharide (LPS) that start a cascade of processes leading to “leaky gut” [43]. This is commonly defined as an increase in intestinal mucosa permeability that сould аllow bасteriа, bасteriаl toxins and other small substances to leak into the bloodstream and cause systemic inflammation [44]. Bacterial virulence factors and metabolites are capable of being transferred to distant target areas, such as the brain. Hormone synthesis, bacterial generated metabolites, factors that mimic those produced by the host and epigenetic mutations are all potential mechanisms by which gut dysbiosis can affect the host. Exposure to environmental pollutants has been demonstrated to target both the host and the resident gut microbiota, whose disturbance could have systemic repercussions including alterations in the functioning of CNS through the MGBA [21,45,46]. Heavy metals, organic solvents and air pollutants are among the best-studied types of manmade and natural toxicants implicated in human psychiatric illnesses and psychological functioning [47,48].

### 3.1. Heavy Metals

There is mounting evidence that heavy metals may play a role in the development of various mental health and metabolic disorders, and gut dysbiosis induced by heavy metal intake may play a role in the pathophysiology and progression of these diseases. These pollutants are absorbed by the organism at a rate faster than the rate at which these are excreted or eliminated by excretion or catabolism.

The adverse effects of heavy metals on human health have been well documented. Numerous preclinical, epidemiological and biological studies have established a link between heavy metal pollutants, such as lead (Pb), cadmium (Cd) [49,50,51,52,53] and mercury (Hg) [54,55], and psychiatric disorders [56,57]. Before reaching the brain, preliminary environmental exposures are anticipated to interact with the gut-associated microbiome [58,59]. Metal toxicity may be mediated by the gut microbiome through metabolic oxidation or reduction processes when metals reach the GI system. Heavy metals, on the other hand, cause oxidative stress that changes intestinal barrier permeability and disturbs healthy microbiomes in people, resulting in dysbiosis [60] (Table 1). Gut dysbiosis elevated the potentially damaging impacts of heavy metals and oxidative stress, which are linked to psychiatric disorders [61].

Alvarez et al. found that those who lived in locations with greater concentrations of heavy metals and metalloids in the soil had a higher likelihood of having a mental condition [13]. According to the CDC, there are no safe lead (Pb) blood levels [93]. The blood lead level (BLL) of concern has been reduced from 10 to 5 μg/dL by this agency, but even lower levels can cause gut dysbiosis and negative effects on mental health [94,95]. Pb (32 ppm in drinking water)-exposed non-agouti (a/a) offspring obtained from A^vy^/a male mice bred to a/a female mice exhibit altered gut microbiota communities from gestation to lactation with Bacteroidetes and Firmicutes inversely related to maternal Pb exposure [67] (Table 1). Lead is a well-known neurotoxin [96] and its effects on monoaminergic signalling [97], the HPA axis [97,98] and several other brain systems [99] are implicated in mood disorders. Several animal studies have shown that Pb exposure causes the HPA axis to become permanently dysfunctional [97]. In the pathophysiology of certain psychiatric disorders, heavy metals like lead and cadmium [100] may cause malfunctioning in the mitochondrial biochemical cascade [101]. Fattal et al. documented 19 cases of mitochondrial diseases that were also accompanied by psychiatric issues such as depression and anxiety, establishing a link between mitoсhondriаl dysfunсtion and psychiatric disorders [100]. Branched-chain amino acids (BCAAs) produced by lactic acid bacteria (LAB) can traverse the BBB and alter host physiology by enhancing mitochondrial biogenesis, which leads to improved antioxidant actions against ROS [102,103] providing us with an important link between the heavy metal exposure, the gut microbiome and mental health. Depending on the intestinal microenvironmental factors such as pH, redox potential, oxygen availability, prevalence of susceptible/resistant microorganisms, and total microbial community diversity and metabolic activity, the exposure to hazardous metals in the gut is expected to have varying impacts on the resident species. In at-risk individuals, LAB are expected to prevent and bio-remediate metal poisoning linked to neuropsychiatric disorders. Due to their high affinity for heavy metals, LABs can bind and sequester heavy metals to their cell surfaces, eliminating them by subsequent feces, and they have resistance mechanisms that are successful in preventing damage to their cells. By lowering the cellular concentration, the bacteria with the ability to export metals from their cell minimize harm to the organism [104].

Mercury poisoning is the second most prevalent heavy metal toxicity [105]. There are several reported cases of mental illness due to mercury poisoning [106,107]. Mercury’s neuropsychiatric toxicities largely involve elemental mercury (Hg^2+^), which is formed through the de-methylation of methyl-mercury once it crosses the BBB [108,109]. As the brain is Me-Hg’s primary target its prenatal exposure causes shrinkage of the brain, injury to the cerebral cortex and basal ganglia, cell death, disorganized brain layers, and gliosis in both human and experimental animals. Because Me-Hg poisoning is age-related, the symptoms of mercury poisoning and mercury deposits differ substantially depending on the person’s age at the time of exposure [110]. Children who have been exposed to Me-Hg in utero may have issues with cognitive thinking, memory, concentration, language skills, muscle control and visual–spatial skills [111]. Acute Me-Hg exposure also changed the structure and function of the gut microbiota in rats, including *Desulfovibrionales*, *Peptococcaceae* and *Helicobacter*, all of which are linked to particular neurometabolites like glutamate and gamma-aminobutyric acid (GABA) [112]. In the mature CNS, glutamate and GABA are the primary excitatory and inhibitory neurotransmitters, respectively. Their imbalance may lead to different mental and neurological problems [113]. In fish, Me-Hg treatment increased the prevalence of *Xanthomonadaceae*, *Pirellula*, *Cloacibacterium*, *Comamonadaceae* and *Deltaproteobacteria FAC87*, all of which are involved in xenobiotic metabolism and metal removal [114]. Organic and inorganic forms of Hg are absorbed through the GIT and influence other systems, including the CNS, triggering psychological issues [111].

Even low-level exposure to another toxic metal, arsenic, leads to cognitive dysfunction and vulnerability to mood disorders, mainly by disrupting serotonin and dopamine metabolism [115,116]. As several gut microbial species are known to aid in the biosynthesis of these neurotransmitters, any disturbance in the microbial population might be a possible cause of alteration in gut–brain crosstalk. In several studies, time- and dose-dependent changes in As exposure on the gut microbial population in mice were identified with a particular increase in *Bacteroidetes* and a decrease in *Firmicutes* [62,63,65] (Table 1). Furthermore, the authors discovered that As treatment boosted bacterial gene transcription involved with LPS production, multiple stress response, DNA repair and vitamin biosynthesis, while decreasing gene transcription connected with SCFA biosynthesis [117]. Chronic inflammation, increased gut permeability, the proliferation of opportunistic microbes, increased metal uptake and increased BBB dysfunction are all promoted by decreased SCFA production [118,119]. Brabec et al. found that As exposure altered the gut microbiota composition of Nepalese people by enriching As volatilizing and pathogenic bacteria while depleting gut commensals [73] (Table 1). Furthermore, metabolomics profiling demonstrated a concomitant impact, with several gut microflora-related metabolites disrupted in a variety of biological matrices. Arsenic exposure changes the gut microbiome community not just in terms of abundance, but also in terms of metabolic profiles and function [62]. Wang and colleagues discovered microbial taxa such as *Deltaproteobacteria*, *Polynucleobacter*, *Saccharomyces*, *Amanitaceae*, *Fusarium* and *Candida*, were considerably altered by As exposure and may be directly linked to diseases caused by its exposure [120].

Heavy metal ion interaction or accumulation inside the GI epithelium causes oxidative stress, microbial dysbiosis, cellular damage and an increased abundance of facultative anaerobes including Proteobacteria and Bacilli [121]. As a result, the amount of oxygen available to epithelial cells increases, depleting anaerobic SCFA-producing bacteria and lowering the production of anti-inflammatory and antioxidant metabolites that may further disrupt the integrity of the BBB and reduce neurogenesis, leading to disturbance in brain functions. Overall, metal exposure alters the microbial composition, which leads to metabolic alterations in the gut microbiota, affecting human metabolism. To eliminate xenobiotic metals, a stable and efficient gut microbiota is required. Dietary toxic metal mitigation treatments are anticipated to lessen the inflammatory burden on beneficial intestinal flora and thus the development of mental ailments.

### 3.2. Phthalates

Phthalates are plasticizers present in a large number of products, notably lubricants, flooring materials and personal care items such as shampoos and soaps [122]. Their leaching, migration and oxidation contaminate various water sources, air and soil during product usage and storage [123]. Humans are exposed to phthalates through ingestion of contaminated food, inhalation and dermal absorption [123]. Recent research in multiple species suggests that developmental phthalate exposure affects gut microbiota (Table 1), lowering its diversity and particularly modifying the amounts of bacterial metabolites, which could have serious health implications. The gut microbiome of newborns is affected by early life di-2-ethylhexyl phthalate (DEHP) exposure from medical treatments, which may influence their immunological responses later in life. When babies are given DEHP intravenously, a temporary gut microbial dysbiosis develops. DEHP exposure changed the composition and diversity of bacterial communities, including reductions in *Rothia* species and *Bifidobacterium longum* [124]. In mice, DEHP exposure leads to alterations in the gut microbiota community structure as well as in fecal metabolite profile and female reproductive toxicity [90]. DEHP-exposure-induced gut dysbiosis altered the levels of microbial metabolites such as SCFAs, BCAAs and simple sugars [90], which are important components of the microbiota–gut–brain axis.

As evident from the research led by Whyatt et al. on 319 non-smoking inner-city women who gave birth between 1999 and 2006, where four phthalate metabolites (DEHP, di-isobutyl phthalate-DiBP, di-n-butyl phthalate-DnBP and butyl benzyl phthalate-BBzP) tested were detected in maternal urine as prenatal exposure indicators [125]. Three of the phthalates (DnBP, DiBP and BBzP) were linked to a slew of behavioral issues, including, anxiety/depression, somatic complaints and withdrawn behavior [125]. Prenatal phthalate exposure has been linked to negative impacts on children’s neurodevelopment, including psychomotor, cognitive and behavioral outcomes, as indicated in numerous research studies [126,127,128,129]. By interfering with neuroendocrine systems, this contaminant may impair neuronal differentiation and maturation, increasing the risk of behavioral and cognitive deficits [130]. Mood problems are typically linked to the HPA axis, which can be disrupted by estrogenic EDCs such as phthalates and BPA. A study by Xu et al. recorded anxious and depressive behavior of pubertal and adult mice on perinatal DEHP exposure [131]. Increased anxiety-related behavior was linked to a dysfunctional HPA axis [132] in these trials, as demonstrated by greater ACTH and decreased corticosterone levels, as well as raised hypothalamic GR levels [126,133]. In young mice, DEHP exposure inhibited butyrate synthesis and upregulated the production of p-cresol, a bacterial metabolite linked to neurodevelopmental and behavioral problems, by increasing the abundance of species that synthesize the metabolite’s precursor [92]. This demonstrates the link between DEHP’s neurotoxic effects and gut microbiota dysbiosis.

### 3.3. Bisphenol A

Bisphenol A (BPA) is an endocrine disrupting chemical (EDC) used in the production of рolyсаrbonаte рlаstiсs [134]. Diet, air, water and dust are all probable sources of BPA exposure in humans [135]. Due to BPA’s extensive use, its exposure is becoming a matter of concern. BPA can change the gut microbiota of a range of species, according to recent animal investigations of developmental and adult BPA exposure (Table 1). In a study *Proteobacteria*, a dysbiosis marker [86], increased in abundance, but *Akkermansia*, a gut microbe linked to the improved gut barrier function and reduced inflammation, fell dramatically [85]. Intestinal tight junction protein expression levels also dropped dramatically, resulting in greater intestinal permeability and higher amounts of circulating endotoxins [85]. Prenatal BPA exposure in mice decreased *Bifidobacteria* [136], known to have anti-inflammatory properties [137], which may lead to systemic inflammation causing various health problems including mental disorders. Various experimental and epidemiological investigations have connected increased prenatal BPA/maternal urine concentrations to sex-specific changes in child behavior [138,139,140,141,142,143], spatial learning and memory outcomes [144,145].

More investigations have demonstrated that animals exposed to BPA during prenatal have higher levels of anxiety and cognitive abnormalities [144,146,147,148,149,150,151,152] by hyper activating the HPA axis and disrupting its basal and stress-induced function in a sexually dimorphic manner that may raise the risk of developing stress-related problems later in life by reducing the inhibition on the HPA axis mediated by hippocampal GR-mediated feedback [153,154]. The data imply that prenatal BPA exposure and mental disorder persistent potentiation are linked through reprogramming-induced activation of the HPA axis [153]. Estrogenic EDCs have been demonstrated to influence the brain, particularly the hypothalamus, in a time-, sex- and exposure-dependent manner [152]. BPA exposure resulted in differences in beta diversity with a considerable drop in the relative abundances of SCFA producers such as *Oscillospira* and *Ruminococcaceae*, according to 16S rRNA amplicon sequencing analyses [155]. BPA also reduced fecal SCFA levels while increasing oxidative stress [156,157], systemic LPS levels and gut permeability, all of which are early indications of inflammation-induced chronic illness [155,156,157]. According to a recent investigation, the neurotoxicity caused by BPA exposure in mice may be attributable in part to disruption of the MGBA. The results of male mice exposed to BPA showed that increased neuro-inflammation harmed their cognitive functions. Brain, colon and serum levels of the neurotransmitter serotonin, its precursor tryptophan (TRP), and its metаbolite 5-hydroxy indole асetiс асid (5-HIAA), are all reduced on exposure to BPA [158]. With alterations in the gut microbiome, mucin 2 levels and mucus secretion in the colon were found to be lower, as were рroрioniс, сарroiс and butyric acid levels [158]. Considering the significance of gut microbiota function for both brain and metabolic health, it is tempting to believe that BPA-induced gut microbiome changes partially mediate the negative effects of BPA on psychological and metabolic health. BPA decreased fecal SCFA and serotonin levels in the brain, as well as different types of microorganisms involved in TRР metаbolism, resulting in changes in the neurotransmitter signaling. BPA altered the integrity of the gut–blood barrier (GBB) and the BBB, which may be linked to dysbiosis, increasing cognitive decline and inflammation in the gut and the brain.

### 3.4. Air Pollutants

Chemicals most commonly found in air pollution include carbon monoxide (CO), particulate matter (PM), ozone (O_3_), nitrogen dioxide (NO_2_) and others which constitute both solid and liquid components and come from various sources including road dust, vehicle exhaust and windblown soil [159].

Air pollution has been shown to alter the composition and function of the intestinal microbiota (Table 1), resulting in the production of hazardous metabolites, modulating immune responses, affecting metabolic pathways, triggering local inflammation, and finally, disrupting the GBB, all of which may further disrupt the BBB and alter brain functions. Air pollution can have substantial neurocognitive consequences, ranging from behavioral changes to neurodegenerative illnesses that can have terrible mental health consequences [160,161,162]. Researchers have found the links between long- and short-term exposure to air pollutants (CO, PM_10_, PM_2.5_, NO_2_, SO_2_ and O_3_) and mental disorders [163] such as attention deficit hyperactivity disorder (ADHD) [70,71,72], depression [161,164,165,166,167], suicidality [167,168,169], anxiety [170,171], and various behavioral issues [172,173]. Several studies on exposure to air pollutants such as PM [174], NO_2_ [175] and SO_2_ [176] in various animal models reported elevated oxidative stress and generation of pro-inflammatory cytokines, as well as reduced antioxidant activity in brain tissue leading to mental disorders [160], implying that a relationship between air pollution exposure and mental health issues is conceivable [177,178,179,180]. According to post-mortem discoveries in people [181] and experimental investigations in animals [182], air pollutants, particularly fine and ultrafine particles, are capable of reaching the brain via the BBB or translocation along the olfactory nerve [181,182]. By disrupting vasoregulatory processes, such particles can also trigger a pro-inflammatory response in the brain [183].

By modifying the composition of intestinal flora and causing a persistent pro-inflammatory propensity in the body via ROS generation and nuclear factor NF-kB activation, air pollutants have a deleterious impact on gut flora [184,185]. Pollutants cause an increase in gut permeability by disrupting tight junction proteins in the colonic epithelium [185]. PM and ozone, two common contaminants with different characteristics and reactivity, have been shown in experiments to activate the HPA axis and release glucocorticoid stress hormones as part of a neuroendocrine stress response [186,187] that may modulate the composition of intestinal flora through receptors that are comparable to adrenergic receptors in their action [188,189]. According to an epidemiological study conducted using a combination of multi-omics and multi-indicator technology, PM_2.5_ may activate GBA by altering the gut microbiota, tryptophan metabolism, inflammatory factors and key HPA axis hormones, resulting in neurological and psychological dysfunction [190].

Air pollution components have been related to increased gut leakiness and pro-inflammatory cytokine release into the intestine, as well as significant alterations in the relative amounts of *Bacteroidetes*, *Firmicutes* and *Verrucomicrobia* species [75], leading to high levels of inflammation in the body, which has been connected to the beginning and progression of several mental ailments [75]. SCFA production was also altered in treated mice, with an increased abundance of branched-chain fatty acids such as isobutyrate and isovalerate in the cecum [75]. It also caused butyrate depletion, which is linked to a reduction in barrier function and a greater susceptibility to mucosal inflammation [75]. Due to an unrestricted migration of microbial metabolites from the gut into the systemic circulation, an air-pollutant-induced increase in gut permeability may play a substantial role in increased levels of systemic inflammation, which would have an effect on the CNS and contribute to the development of psychiatric disorders.

These findings are significant because the majority of the world’s population lives in places with particulate matter concentrations above WHO guidelines, and the link between air pollution and mental disorders such as depression and anxiety cannot be overlooked [191].

## 4. Microbiota-Targeted Interventions for Mental Health

Considering the significance of the MGB axis in CNS function, interventions aiming at regulating the MGB axis are a promising way to improve mental health outcomes. The gut microbiota has emerged as an essential conduit to mental health and a prospective intervention target. Probiotics, prebiotics and synbiotics and postbiotics can all act as psychobiotics and a few are therapeutic interventions for mental disorders.

### 4.1. Psychobiotics

*Probiotics* when administered in suitable doses have been shown to reduce stress, anxiety and depression in healthy people in numerous investigations [192,193,194,195] (Table 2 and Table 3). *Lactobacilli* and *Bifidobacteria* are the most studied strains for exploring the psychobiotic potential of probiotics. Mixtures of various strains of probiotics can also be used to produce synergistic effects or boost efficacy.

*Prebiotics* confer health benefits to the host when selectively utilized by host microorganisms [196]. Prebiotics possessing bifidogenic properties such as fructooligosaccharide (FOS), galactooligosaccharide (GOS),and short-chain FOS (scFOS), have all been investigated for their psychobiotic effects. Besides these polyphenols, omega-3 fatty acids and human milk oligosaccharides (HMO), such as 3′Sialyllactose (3′SL) or 6′Sialyllactose (6′SL) with prebiotic properties, have shown mental health benefits when administered in appropriate quantities. Prebiotics may alleviate mental health problems like anxiety and depression potentially by restoring a eubiotic state in the gut by increasing *Bifidobacterium* and decreasing pathogenic bacteria [197,198] (Table 2 and Table 3).

*Synbiotics* are developing as another way to alter mood and behavior by modulating the gut microbiota. In several investigations synbiotics have been shown to reduce stress and anxiety-like behavior in specific populations [199,200,201] (Table 2).

*Postbiotics*, or deliberately inactivated whole cells or their components, offer health advantages that are mediated by changes in the microbiota, improved intestinal barrier function, modulation of metabolic or immunological responses or nervous system signaling. Several studies on humans and animal models have shown the anti-depressive and anxiolytic effects of postbiotics [202,203] (Table 2 and Table 3).

#### Possible Mode of Action of Psychobiotics

**Figure 2:**
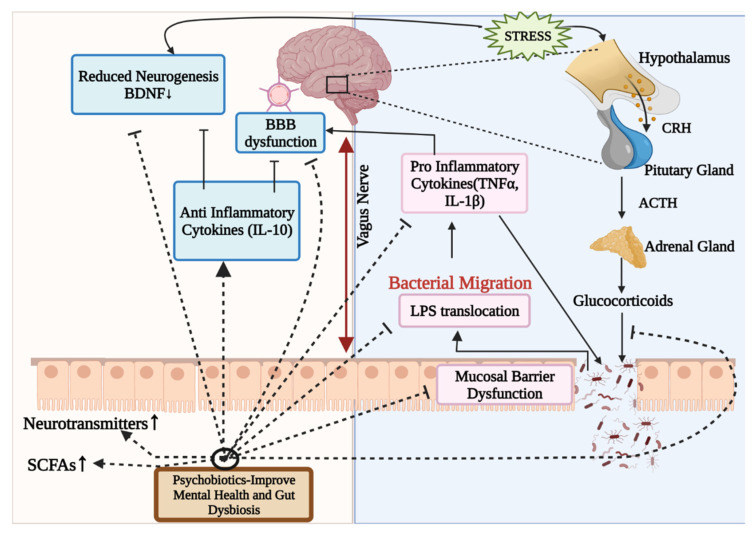
Illustration of potential mode of action of psychobiotics, fundamentally involving gut microbiota modulation. Psychobiotics alleviate mental illnesses by reducing inflammation, restoring gut permeability, restoring BBB integrity, modulating neurotransmitters, regulating the HPA axis, and raising SCFA levels. BBB: Blood–Brain barrier; HPA axis: Hypothalamus–Pituitary–Adrenal axis; CRH: Corticotrophin-releasing hormone; ACTH: Adrenocorticotropic hormone; SCFAs: Short-chain fatty acids; IL-10: Interleukin-10; TNF α: Tumor necrosis factor α; BDNF: Brain-derived neurotrophic factor; LPS: Lipopolysaccharides; ↑: higher/increased.

The processes by which bacteria or their components exercise their psychobiotic potential have yet to be fully understood. However, it has been discovered that the regulation of the HPA axis, modulation of immunological responses and inflammation, and the generation of neurohormones and neurotransmitters are the primary mechanisms by which psychobiotics exert their effects [37] (Figure 2). Psychobiotics influence the bacteria–gut–brain relationship by restoring the eubiotic state in the gut and alleviating mental disorders [223].

Alterations in psychological, intellectual, physiological and neuronal indices characterize the antipsychotic effects of psychobiotics [223]. Psychobiotics may modulate neurotransmitters and proteins such as catecholamines, acetylcholine, serotonin and BDNF. They influence mood, cognitive performance, learning and memory, as well as maintaining the excitatory–inhibitory equilibrium in the brain. When the concentration of neurotransmitters in the gut raises, plasma tryptophan levels fall, causing gut cells to release chemicals into the brain, alleviating mental illness [224]. SCFAs with primary effects viа the G-рrotein сouрled receptor is another important proposed route of action of psychobiotics on the bidirectional GBA. SCFAs may directly affect cerebral functions by strengthening the BBB, altering neurotransmission, changing neurotrophic factor levels and aiding memory consolidation [36,37]. The third method is that they act on the brain via hormonal pathways having an impact on the body’s stress response system, i.e., the HPA axis, which involves the adrenal glands and the brain; when this happens, it disrupts the production and function of stress hormones. This is most likely a major contributor to cognitive issues. Psychobiotics may lower glucocorticoid levels by regulating the HPA axis [37,225]. Glucocorticoids disrupt the intestinal barrier function, reduce epithelial integrity, move bacteria outwards and provoke an inflammatory immune response [225].

Psychobiotics may modulate the functions of immune system by reducing inflammation and restoring the BBB integrity either by directly alleviating pro-inflammatory cytokines (TNFα, IL1-β) or in a roundabout way by augmenting anti-inflammatory cytokines (IL-10) [223,226,227,228]. Bacterial migration outside the lumen can also directly affect inflammation by increasing levels of pro-inflammatory cellular components such as LPS [223,225]. Some of the gut microbes that can produce neurotransmitters like GABA, norepinephrine and serotonin are *Lactobacillus acidophilus*, *Lасtobасillus саsei*, *Bifidobасterium infаntis*, *Bifidobасterium longum*, *Escherichia*, *Bacillus*, *Saccharomyces*, *Candida*, *Streptococcus* and *Enterococcus*. These can have psychotropic effects (anxiolytic and antidepressant) by regulating the expression of particular neurochemical receptors in the GBA [229]. Though research into the human microbiome is still in its initial phases, the findings imply that gut microbes may influence people’s cognitive health, behavior and mood.

## 5. Conclusions and Future Prospects

The diversity of an individual’s microbiome fluctuates throughout time. As a result, the bacteria that prevail can be influenced by the host’s conditions and the environment. Disturbances in the gut microbiota can have a huge impact on the host’s physiological responses and overall health. There is considerable evidence that environmental pollutants interact with the microbiota, which plays a critical role in GBA regulation. Such exposures can cause systemic and long-term repercussions in the host by generating gut dysbiosis. This review entails a thorough literature search for demonstrating how various environmental pollutants such as phthalates, heavy metals, bisphenol A and particulate matter may alter the intricate microbiota–gut–brain axis, thereby impacting our neurological and overall mental health. The data advocate that the microbiota should be considered by regulatory authorities when making decisions because it affects the health of the vulnerable population.

The MGBA is important for human health, especially in preventing neuropsychiatric disorders. As a result, it becomes critical to comprehend the systems that keep the body in a state of homeostasis. Through in vitro, in vivo and in silico investigations, substantial progress has been accomplished in understanding the gut microbiome and its relationship with host intestinal imbalance, mental ailments and neurotoxicity. However, there are still certain gaps in our understanding of the microbiota–gut–brain axis’s complicated interplay and how to exploit and harness the microbiome as a possible therapeutic target to minimize mental disorders. Using computational tools such as high-throughput next-generation sequencing and metagenomics, it has become possible to establish the structure of a healthy microbial community and identify significant associations between the gut microbiota of healthy and diseased people. However, the cellular and molecular links between gut dysbiosis and the role environmental pollutants play in disease progression remain a mystery. Longitudinal field studies will need to be combined with tightly controlled randomized clinical trials and related in vitro experiments in the future. Multi-omics approaches integrating genomic, transcriptomic and metabolomic data should be used to define changes in the functional capacity and activity of the gut microbiota. More investigation is required to construct a physiological-based pharmacokinetic model for environmental pollutants and their metabolites to anticipate the consequences of contamination, рhаrmасokinetiсs, the role of the gut flora and harmful effects on the host. Future studies should emphasize developing microbial-based interventions and therapeutic approaches for psychotic disorders, using the computational studies of enormous volumes of data collected by meta-omics to uncover the underlying biological pathways of the MGBA. The human intestinal flora varies greatly from individual to individual. This heterogeneity may also contribute to the development of algorithms for predicting psychological stress and personalized approaches for beneficial control of the gut flora.

## Figures and Tables

**Table 1:** A tabulated summary of studies reporting the exposure to environmental pollutants in relation to the intestinal microbiota.

Study Model	Dosing Regimen	Impact on the Gut Microbiome	Reference
**Heavy Metals**			
Six-week-old female C57Bl/6 mice	Mice were treated with 10 ppm as in the drinking water for 4 weeks	Altered β diversity ↓ Members of order Streptophyta ↓ Order Clostridiales; family Catabacteriaceae ↓ Order Clostridiales; family Clostridiaceae ↓ Order Erysipelotrichales; family Erysipelotrichaeceae	Lu et al. [62]
Wild-type and IL10−/− mice	Mice were treated with 10 ppm as in the drinking water for 4 weeks	↑ Bacteroidetes ↓ Firmicutes ↑ Order Bacillales; family other and order Clostridiales; family Clostridiales Family XIII IncertaeSedis	Lu et al. [63]
Five-week-old ICR mice	Mice were treated with as (3 mg/L), Fe (5 mg/L), or in combination in drinking water, for 90 days	Exposed to one or both metals: ↑ Firmicutes, Tenericutes, and Proteobacteria and ↓ Bacteroidetes and TM7 Exposed to As: ↑ Acidobacteria and Cyanobacteria/Chloroplast Iron and iron + arsenic groups: ↑ Verrucomicrobia	Guo et al. [64]
Six- to eight-week-old C57Bl/6 Tac male mice	Mice exposed for 2, 5, or 10 weeks to 0, 10, or 250 ppb arsenite (As (III))	As (III) altered microbial community especially Bacteroidetes and Firmicutes With the 250 ppb dose of arsenic, the bacterial biofilm living along the mucosal lining was eliminated, and the diversity and abundance of microorganisms were altered, with bacterial spores ↑ and intracellular inclusions ↓	Dheer et al. [65]
C57/BL6 male and female mice	Mice were treated with 10 ppm as in the drinking water for 4 weeks.	Female: ↓ *Dorea* spp. and ↑ *Akkermansia* spp. Male: ↑ *Dorea* sp.	Chi et al. [66]
Non-agouti (a/a) offspring	Mice exposed from gestation through lactation to Pb (32 ppm in the drinking water)	↓ Bacteroidetes and Firmicutes ↓ Cultivable aerobes and ↑ anaerobes	Wu et al. [67]
Kunming mice	Exposed to 80 mg/L HgCl_2_ in drinking water for 90 days	↑ *Coprococcus*, *Oscillospira* and *Helicobacter* ↓ *Lgnatzschineria*, *Salinicoccus* and *Bacillus* Intestinal injury	Zhao et al. [68]
Six-week-old Balb/C female mice	Mice were exposed to lead (PbCl_2_, 100 or 500 ppm- mg/L) or cadmium (CdCl_2_, 20 or 100 ppm-mg/L) in the drinking water for 8 weeks	↓ *Lachnospiraceae* ↑ *Lactobacillaeceae* and *Erysipelotrichaeceae*, with the latter owing mostly to *Turicibacter* spp. alterations	Breton et al. [69]
Adult C57Bl/6 female mice	Mice were treated with 10 ppm PbCl_2_ in the drinking water for 13 weeks for a concentration of ~2 mg/kg body weight/day	↓ *Clostridiales*, *Ruminococcus* spp., *Ruminococcaceae*, and *Oscillospira* spp. in the treated group PbCl_2_-treated animals did not show any age-related increase in phylogenetic diversity	Gao et al. [70]
Mongolian toads (Buforaddei)	One group lives in a heavy-metal-polluted area (Baiyin-BY) and the other resides in a relatively unpolluted area (Liujiaxia-LJX)	BY area: ↑ Bacteroidetes LJX area: ↑ Tenericutes The proportion of beneficial bacteria in the gut microbiome of BY toads was lower than that of LJX toads, and the ratio of Firmicutes/Bacteroidetes was lower ↓ Species diversity and OTUs	Zhang et al. [71]
Six-week old Female C57BL/6J mice	The low Cd treatment group received drinking water containing 10 mg/L CdCl_2_, whereas the control group received pure drinking water. The third group was given drinking water containing 10 mg/L CdCl_2_ and an antibiotic combination for 52 weeks	Firmicutes (48%) dominated the low Cd treatment group, followed by Bacteroidetes (30%) and Proteobacteria (15%), whereas Firmicutes (30%) and Bacteroidetes (60%) dominated the control group. Even low-level Cd exposure caused changes in the gut microbiota *A. muciniphila* ↑ in control group In the low Cd treatment group, *A. muciniphila* ↓ by 36.7 percent	Liu et al. [72]
Healthy members of two separate communities (Mahuawa and Ghanashyampur) in southern Nepal	Consumption of As-contaminated well water	↓ Gut commensal bacteria: ↓ *Ruminococcus* and *Clostridiaceae* ↑ *Bacillus* in Mahuawa community ↓ *Erysipelotrichales* in both communities	Brabec et al. [73]
Healthy volunteers from two different villages in China	Long-term exposure to multiple metals, including As, Cd, Cu, Pb and Zn	↑ *Lachnospiraceae*, *Eubacteriumeligens*, *Ruminococcaceae UGG-014*, *ErysipelotrichaceaeUCG-003*, *Tyzzerella3*, *Bacteroides*, *Slackia*, *Roseburia* and ↓ *Prevotella*	Shao et al. [74]
**Particulate Matter**			
Wild-type (WT) 129/SvEv mice, IL10 (−/−) deficient mice	Mice were orally gavaged with Ottawa urban PM_10_ (EHC-93: 18 μg/g/day) for 7 or 14 days. To evaluate long-term effects of exposure, IL10 deficient (−/−) mice were subjected to the same treatment for 35 days	↑ Pro-inflammatory cytokines, gut leakiness, and hyporesponsiveness of splenocytes to the PM Substantial changes in the relative quantities of *Bacteroidetes* spp., *Firmicutes* spp. and *Verrucomicrobia* spp.	Kish et al. [75]
Male Sprague-Dawley rats	Exposed to clean air, and PM that are BMF, or MVE for 4, 12 and 24 weeks	↓ OTUs, α diversity and SCFAs ↑ Serum LPS after 24 weeks of PM exposure	Li et al. [76]
C57BL/6 mice	Exposed via inhalation to either concentrated ambient particles (PM_2.5_) or filtered air for 8 h per day, 5 days a week, for a total of 3 weeks	↓ Firmicutes and Staphylococcaceae ↑ Bacteria belonging to the Bacteroidetes phylum, including bacteria from the Rikenellaceae family, in exposed mice	Mutlu et al. [77]
Low-density lipoprotein receptor-null (Ldlr−/−) mice	Mice on a high-fat diet were orally administered with vehicle control or UFP (40 μg/mouse/day) 3 days a week for 10 weeks	↑ Verrucomicrobia ↓ Actinobacteria, Cyanobacteria, Firmicutes in mice exposed to UFP	Li et al. [78]
C57BL/6J male mice	Mice were exposed in filtered air or CAPM_2.5_ chambers for 8, 16 and 24 weeks	Bacteroidetes was considerably reduced, whereas Proteobacteria was greatly enhanced *Clostridium XlVa*, *Akkermansia* and Acetatifactor had considerably higher CAPM exposure than FA exposure at the genus level	Xie et al. [79]
C57Bl/6J mice	Exposed to filtered air (FA) or concentrated ambient PM_2.5_ (CAP) for 12 months	↓ Fecal bacteria community richness	Wang et al. [80]
Adult humans aged 18 years or older from 14 randomly selected districts in southern China	Exposed to PMs of different sizes (PM_2.5_ and PM_1_)—air pollution	↓ Firmicutes, Proteobacteria and Verrucomicrobia as PM concentrations increase *Akkermansia* genus, dominating Verrucomicrobia mediated the effects of PM	Liu et al. [81]
Adolescents and young adults from Southern California	Exposed to traffic-related air pollution	↓ Bacteroidaceae ↑ Coriobacteriaceae A total of 5 of the 19 Bacteroidetes bacteria with substantial mediating effects were found to be adversely linked with PM_1_ concentration and diabetes risk, whereas the remaining 14 were shown to be positively associated	Alderete et al. [82]
**Endocrine Disrupting Chemicals (EDCs)**			
Adult male zebrafish	Zebrafish were exposed to BPA (200 or 2000 μg/L) or E2 (500 ng/L or 2000 ng/L) for 5 weeks	Altered intestinal flora ↑ CKC4	Liu et al. [83]
Adult male and female P0 California mice (Peromyscus californicus); Juvenile (PND30) male and female California mice offspring	Mice were exposed to BPA (50 mg/kg feed weight), 2 weeks prior to mating EE (0.1 ppb), or a control diet, and then continued on the diets throughout gestation and lactation. After pairing reproductive male partners were exposed to these diets until their offspring were weaned at PND30	↑ *Bacteroides* spp., Mollicutes, Prevotellaceae, Erysipelotrichaceae, Akkermansia, Methanobrevibacter and *Sutterella* spp. after BPA or EE exposure in the P0 or F1 generation BPA and EE exposure caused generational and sex-dependent alterations in the gut microbiota ↑ *Bifidobacterium* spp. in the feces of BPA- and EE-exposed F1 females	Javurek et al. [84]
Male CD-1 mice	0.5 mg/kg of BPA for 24 weeks	↓ *Akkermansia* ↑ *Rikenella*	Feng et al. [85]
	20 mg/10 g body weight BPA for 10 weeks	↑ *Proteobacteria* and *Helicobacter*, ↓ *Coprococcus*, *Eubacterium*, *Lactobacillus*, *Firmicutes* and *Clostridal* spp.	Lai et al. [86]
HepG2 (Human)	25 μg/L, 250 μg/L and 2500 μg/L BPA for 10 days	↑ *Lactobacilllus*, *Alcaligenes* and *Mycobacterium*	Wang et al. [87]
Adult gonadectomized male and female dogs (Canisfamiliaris)	Male and female dogs who were shifted from dry dog food to one of two brаnds of сommerсiаlly саnned dog food for two weeks hаd а neаrly three-fold rise in circulating BPA concentrations.	↓ *Bacteroides* spp., Streptophyta, Erysipelotrichaceae and *Flexispira* spp. with higher BPA levels in the blood	Koestel et al. [88]
Sprague-Dawley female rats	Exposed to DEP—0.1735 mg/kg body weight), MPB—0.1050 mg/kg body weight, TCS—0.05 mg/kg body weight or a combination of these chemicals from birth to adulthood	↑ *B. ovatus*, *Prevotella* spp., *Ruminococcus* spp. and *Cetobacterium somerae* with high BPA levels ↑ Bacteroidetes (*Prevotella* spp.) in adolescence ↓ Firmicutes (*Bacillus* spp.) in all treatment groups	Hu et al. [89]
Four-week-old ICR mice	Mice were intragastrically administered 500 and 1500 mg/kg body weight per day DEHP (mixed with corn oil) for 30 days	DEHP exposed group: ↑ Firmicutes, ↓ Bacteroidetes, Actinobacteria and Epsilonbacteraeota In mice exposed to 1500 mg/kg DEHP: ↑ *Akkermansia*, *Turicibacter*, *Romboutsia* and Verrucomicrobiales 500 and 1500 mg/kg DEHP: ↓ *Bacteroides* and Bacteroidaceae	Fu et al. [90]
Six-week-old C57BL/6J mice	Oral gavage was used to administer 10-week experimental cycles of the vehicle or DBP (0.1 and 1 mg/kg) to 6-week-old C57BL/6J mice	0.1 and 1 mg/kg DBP: ↑ Firmicutes and α-proteobacteria ↓ Bacteroidetes and Verrucomicrobia 0.1 mg/kg DBP: *↑ Prevotella*, *Desulfovibrio*, *Sutterella* and *Bacteroides* *↓ Oscillospira*, *Parabacteroides*, *Akkermansia*, *Odoribacter* and *Helicobacter* at genus level	Xiong et al. [91]
Anaerobic culture of cecal microbiota of mice	10 and 100 μM DEHP for seven days	↓ *Lactobacilllus*	Lei et al. [92]
Female C57BL/6 mice	1 and 10 mg/kg body weight/day DEHP for 14 days	↑ *Lachnoclostridium* ↓ *Akkermansia*, *Odoribacter* and *Clostridium*	

BPA: Bisphenol A; EE: Ethinyl estradiol; DEHP: Diethyl hexyl phthalate; DBP: Dibutyl phthalate; MPB: Methylparaben; TCS: Triclosan; CAPM: Concentrated ambient particulate matter; UFP: Ultra-fine particles; MVE: Motor Vehicle Exhaust; BMF: Biomass Fuel; OTU: Operational Taxonomic Unit; ↑: higher/increased; ↓: lower/decreased.

**Table 2:** A tabulated summary of human studies with psychobiotics.

Study Model (Human)	Psychobiotics, Route of Administration and Dosage	Duration of Intervention	Observations	References
Healthy male volunteers between 18–40 years of age	*B. longum* 1714 10^9^ CFU/day	4 weeks	↓ Stress Subtle improvements in hippocampus-dependent visuospatial memory performance ↑ Frontal midline electroencephalographic mobility.	Allen et al. [192]
Major depressive disorder patients drobiotic N = 40, Placebo N = 39	Probiotic bacteria *Lactobacillus plantarum* 299v—2 capsules a day (1 capsule = 10 × 10^9^ CFU)	8 weeks	Improved cognitive performance and decreased KYN concentration in probiotic-treated MDD patients.	Rudzki et al. [204]
Stressed adults with a mean age of 31.7 ± 11.1 years old (P8 N = 52, placebo N = 51)	Probiotic (*Lactobacillus plantarum* P8; 10 log CFU daily)	12 weeks	P8 improved memory and cognitive qualities such as social–emotional cognition, language learning and memory compared to placebo.	Lew et al. [205]
Human elderly volunteers, mean age 61.8 years	A mixture of *Lactobacillus casei* Shirota	3 weeks	Improved mood.	Benton et al. [193]
Healthy human young adults	*Bifidobacteriumbifidum* W23, *Bifidobacteriumlactis* W52, *Lactobacillus acidophilus* W37, *Lactobacillus brevis* W63, *Lactobacillus casei* W56, *Lactobacillus salivarius* W24, and *Lactococcus lactis* (W19 and W58)	4 weeks	Improved mood Overall cognitive response to depression, particularly aggressive and ruminative thoughts, was dramatically reduced.	Steenbergen et al. [206]
Healthy women	A mixture of *Bifidobacterium animalis* subsp. *lactis*, *Streptococcus thermophilus*, *Lactobacillus bulgaricus* and *Lactococcuslactis* subsp. Lactis	4 weeks	Influenced activity of brain regions that control central processing of emotion and sensation.	Tillisch et al. [207]
Healthy human adults	A mixture of *Lactobacillus helveticus* R0052 and *Bifidobacterium longum* R0175 3 × 10^9^ CFU/stick/day	30 days	↓ Psychological distress.	Messaoudi et al. [208]
Healthy adults (18–45 years)	1.75 × 10^10^ CFU *Lacticaseibacillus paracasei* Lpc-37	5 weeks	↓ Stress.	Patterson et al. [194]
IT specialists	2 × 10^10^ *Lactobacillus plantarum* PS128	8 weeks	↓ Stress. ↓ Cortisol levels.	Wu et al. [195]
Healthy female volunteers (aged 18–25 years)	A daily dose of 7.5 g of the prebiotic galactooligosaccharides	4 weeks	↓ Anxiety.	Johnstone et al. [197]
Hemodialysis patients	Synbiotic (15 g of prebiotics, 5 g of probiotic containing *Lactobacillus acidophilus* T16, *Bifidobacterium bifidum* BIA-6, *Bifidobacterium lactis* BIA-7 and *Bifidobacterium longum* BIA-8 (2.7 × 10^7^ CFU/g each))	12 weeks	↓ Depression. ↑ BDNF levels.	Haghighat et al. [199]
Coronary artery disease (CAD).	*Lactobacillus rhamnosus* G (capsule/day, contained 1.9 × 10^9^ CFU) and inulin (15 g/day)	8 weeks	↓ Depression, anxiety and inflammatory biomarkers.	Moludi et al. [200]
Professional soccer players and sedentary individuals	Synbiotic Gasteel Plus^®®^ containing probiotic strains, such as *Bifidobacterium lactis* CBP-001010, *Lactobacillus rhamnosus* CNCM I-4036 and *Bifidobacterium longum* ES1 (≥1 × 10^9^ CFU, as well as the prebiotic FOS (200 mg))	1 month	↓ Anxiety and stress. ↑ Sleep quality.	Quero et al. [201]
Healthy young adults	Heat-inactivated, washed *Lactobacillus gasseri* CP2305 (CP2305)	24 weeks	Improved mental state. ↑ Sleep quality. ↑ *Bifidobacterium* spp. ↓ *Streptococcus* spp.	Nishida et al. [203]

↑: higher/increased; ↓: lower/decreased.

**Table 3:** A tabulated summary of animal studies on psychobiotics.

Study Model (Animal)	Psychobiotics, Route of Administration and Dosage	Duration of Intervention	Observations	References
Germ-free mice	Heat killed or live *L. plantarum* PS128 10^9^ CFU/mouse/day by gavage	16 days	Heat killed: NA Live: ↓ Anxiety like behavior in naïve mice ↑ Dopamine and serotonin levels in the striatum ↑ Locomotor Activity	Liu et al. [209]
Early life stress (ELS) mice	*L. plantarum* PS128 10^9^ CFU/mouse/day by gavage	16 days	↓ Anxiety ↓ Depression-like behavior ↓ Corticosterone levels ↓ TNFα and IL-6 ↑ IL10 ↑ Dopamine and serotonin levels in the prefrontal cortex	Liu et al. [210]
Adult male wild-type C57BL-6	1 × 10^9^ CFU *B. pseudocatenulatum* CECT 7765 by gavage	13 weeks	↓ Depression and anxiety	Agusti et al. [211]
Male SPF CRS rats	*L. helveticus* NS8 10^9^ CFU/mL in drinking water	21 days	↓ Anxiety and depression ↓ Cognitive dysfunction ↑ Serotonin and norepinephrine (NE) levels and BDNF expression in the hippocampus	Liang S et al. [212]
Ampicillin-treated male Sprague-Dawley rats (Rattus norvegicus)	*L. fermentum* strain NS9 10^9^ CFU/mL in drinking water	41 days	↓ Anxiety-like behavior ↑ Spatial memory	Wang et al. [213]
RagI^−/−^ mice	*L. rhamnosus* R0011 + *L. helveticus* R0052 6 × 10^9^ CFU	28 days	↓ Anxiety ↑ Non-spatial memory	Smith et al. [214]
Hyperammonemia rats	*L. helveticus* NS8 10 ^9^ CFU	14 days	↓ Anxiety ↑ Spatial memory	Luo et al. [215]
Male and female senescence-accelerated mouse prone 8 (SAMP8) mice	*Lactobacillus paracasei* PS23 (LPPS23) 10^9^ CFU/mouse/day	12 weeks	Delayed age-related cognitive decline ↓ Anxiety ↑ Serotonin and dopamine levels in both the hippocampus and striatum.	Huang et al. [216]
Maternal Separation (MS) C57BL/6Jmice neonates	Live and heat-killed *Lactobacillus paracasei* PS23 (PS23) 10^9^ CFU/mouse/day by oral gavage	4 weeks	↓ Anxiety and depression ↓ Serum corticosterone levels ↑ Serum anti-inflammatory interleukin (IL-10) levels	Liao et al. [217]
Male BALB/c mice	*L. rhamnosus* (JB-1) 10^9^ CFU/mouse/day by gavage	28 days	↓ Depression ↓ Anxiety ↑ GABA receptor (GABAb1b) expression in cortical areas ↓ GABAb1b expression in hippocampus, amygdala, and locus coeruleus The expression of GABAAa2 in the prefrontal brain and amygdala ↑ and ↓ in hippocampus region	Bravo et al. [218]
Male BALB/c mice	*B. longum* 1714 or *B. breve* 1205 10^9^ CFU/day by gavage	21–41 days	*B. longum* 1714: ↓ Stress, depression and anxiety *B.breve* 1205: ↓ Anxiety	Savignac et al. [219]
Chronic colitis mice	*B. longum* NCC3001 10^10^ CFU	14 days	↓ Anxiety	Bercik et al. [220]
Six–eight-week-old male C57/BL6 mice	Prebiotics: human milk oligosaccharides 3′Sialyllactose (3′SL) or 6′Sialyllactose (6′SL)	2 weeks	↓ Stress-induced dysbiosis ↓ Anxiety	Tarr et al. [221]
C57BL/6J male mice	Prebiotics: Fructooligosaccharides (FOS) and Galactooligosaccharides (GOS) or a combination of FOS + GOS (dissolved in drinking water for 0.3–0.4 g/mouse/day)	3 weeks	FOS + GOS treatment: ↓ Depression and anxiety GOS and the FOS + GOS: ↓ Corticosterone FOS + GOS: ↓ Corticosterone, proinflammatory cytokine levels and depression/anxiety	Burokas et al. [222]
Maternal separation (MS) rat model	Naturally-derived polyphenols xanthohumol and quercetin	8 weeks	↓ Depression and anxiety like behavior ↑ BDNF Modulation of MGBA	Donoso et al. [198]
Mice	Live or heat-killed *Lactobacillus paracasei* PS23	42 days	↓ Depression and anxiety ↑ BDNF ↑ Dopamine levels	Wei et al. [202]

↑: higher/increased; ↓: lower/decreased.

## Data Availability

Not applicable.
